# Impaired brain homeostasis and neurogenesis in diet-induced overweight zebrafish: a preventive role from *A. borbonica* extract

**DOI:** 10.1038/s41598-020-71402-2

**Published:** 2020-09-02

**Authors:** Batoul Ghaddar, Bryan Veeren, Philippe Rondeau, Matthieu Bringart, Christian Lefebvre d’Hellencourt, Olivier Meilhac, Jean-Loup Bascands, Nicolas Diotel

**Affiliations:** 1 INSERM, UMR 1188, Diabète athérothrombose Thérapies Réunion Océan Indien (DéTROI), Université de La Réunion, Saint-Denis de La Réunion, France; 2 CHU de La Réunion, Saint-Denis, France

**Keywords:** Neuroscience, Physiology

## Abstract

Overweight and obesity are worldwide health concerns leading to many physiological disorders. Recent data highlighted their deleterious effects on brain homeostasis and plasticity, but the mechanisms underlying such disruptions are still not well understood. In this study, we developed and characterized a fast and reliable diet-induced overweight (DIO) model in zebrafish, for (1) studying the effects of overfeeding on brain homeostasis and for (2) testing different preventive and/or therapeutic strategies. By overfeeding zebrafish for 4 weeks, we report the disruption of many metabolic parameters reproducing human overweight features including increased body weight, body mass index, fasting blood glucose levels and liver steatosis. Furthermore, DIO fish displayed blood–brain barrier leakage, cerebral oxidative stress, neuroinflammation and decreased neurogenesis. Finally, we investigated the preventive beneficial effects of *A. borbonica*, an endogenous plant from Reunion Island. Overnight treatment with *A. borbonica* aqueous extract during the 4 weeks of overfeeding limited some detrimental central effects of DIO. In conclusion, we established a relevant DIO model in zebrafish demonstrating that overfeeding impairs peripheral and central homeostasis. This work also highlights the preventive protective effects of *A. borbonica* aqueous extracts in DIO, and opens a way to easily screen drugs aiming at limiting overweight and associated neurological disorders.

## Introduction

Obesity and overweight are defined as excessive body weight characterized by body fat accumulation and could be easily estimated by calculating the body mass index (BMI)^1^. Both obesity and overweight are among the main health concerns worldwide. Their prevalence is increasing annually in developing and developed countries and has nearly tripled since 1980s according to the World Health Organization (2019).

Overweight and obesity are due to an imbalance between energy intake, storage, and expenditure including interactions with hereditary and environmental factors^2–4^. They result in numerous metabolic disorders such as dyslipidemia, non-alcoholic fatty liver, hyperglycemia, insulin resistance, and are characterized by chronic inflammation and oxidative stress^5–7^. These pathologies lead to many physiological disorders such as cardiovascular complications as well as the development of type 2 diabetes and contribute to increased morbidities^7,8^.

In addition to impair peripheral metabolism and homeostasis, overweight and obesity could have a negative impact on central nervous system (CNS) homeostasis, leading to cognitive impairments and dementia^9^. Such cognitive defects have been reported in many animal models such as in high fat diet (HFD)- treated rodents displaying hippocampal-dependent cognitive impairments^10,11^. Among factors contributing to these cognitive dysfunctions, inflammatory and oxidative stress appear as key players leading to blood–brain barrier (BBB) leakage through decreased expression of tight junction proteins in the hippocampus^12^. Other studies have also shown that HFD/DIO could impair brain plasticity such as neurogenesis, a process involved in memory^9^. Although some links between obesity and CNS disruptions have been highlighted, the mechanisms whereby it adversely disturbs brain homeostasis remain unclear.

In order to investigate the impact of metabolic disorders on the CNS, zebrafish recently emerged as an interesting model. Firstly, it is a relevant organism for studying overweight/obesity^13–17^, hyperglycemia^18–21^ and diabetes^22,23^. Secondly, in contrast to mammals in which adult neurogenesis is restricted to two main regions, adult zebrafish display an important number of neurogenic niches throughout the brain^24^. Such neurogenic capacities are relied on the maintenance of numerous neural stem cells during adulthood, namely radial glial cells, as well as further committed progenitors allowing to easily study neurogenesis^25–28^.

Given the health threats due to overweight and obesity, it becomes crucial to attempt to reverse these deleterious effects by preventive and/or therapeutic strategies. Many endemic plants from Reunion Island, a biodiversity hot-spot localized in the Mascarene Archipelago, are traditionally used for their anti-inflammatory, anti-oxidant, anti-diabetic and weight-loss properties. From these plants, 22 were recently registered to the French Pharmacopeia^29–32^. However, scientific data demonstrating the real beneficial effects of these plants in vitro and in vivo are lacking. *Antirhea borbonica* (*A. borbonica*) could be envisioned as an interesting candidate according to its reported anti-inflammatory, anti-oxidant and anti-diabetic effects^29,32,33^.

In this work, we aimed at setting up a fast and reliable zebrafish model of overweight (DIO) by overfeeding fish for 4 weeks in order to investigate the impact of overfeeding on (1) peripheral metabolic parameters (i.e. body weight, body mass index, fasting blood glucose, liver steatosis) and on (2) brain homeostasis focusing on the BBB, neuroinflammation and oxidative stress as well as on neurogenesis. Finally, we aimed at investigating the possible beneficial effects of *A. borbonica* aqueous extract administration in preventing peripheral and central impairments induced by DIO.

## Results

### DIO models induces phenotypic and metabolic changes

**Figure 1:**
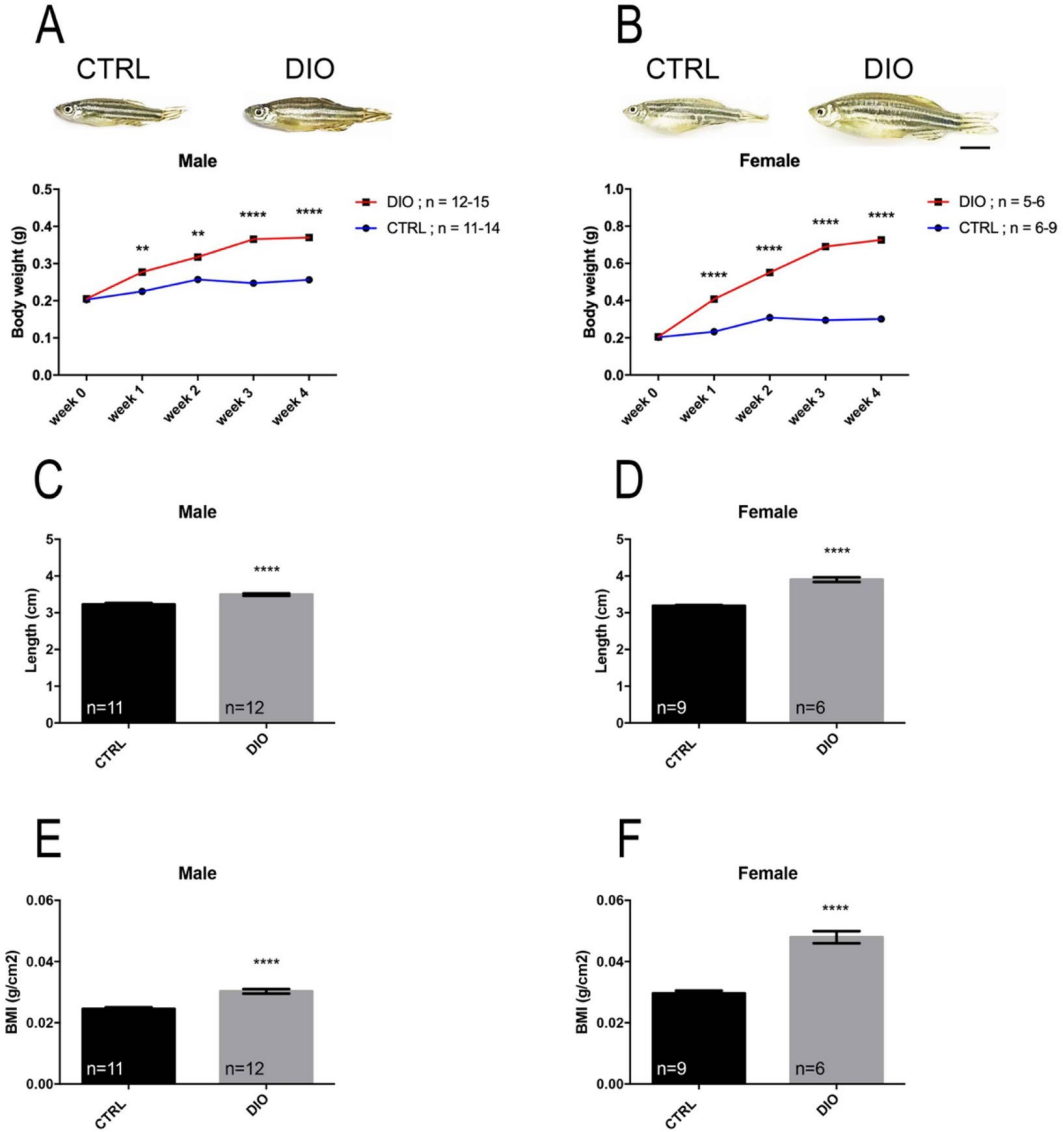
DIO results in increased body weight, length and BMI in both male and female zebrafish. (**A**), (**B**) Graphs illustrating the body weight measurements during 4 weeks for both CTRL and DIO-treated zebrafish in male and female, respectively. The zebrafish pictures highlight the morphological differences at week 4. (**C**), (**D**) Body length measurements at week 4 in male and female zebrafish, respectively. (**E**), (**F**) Body mass index (BMI; grams per square centimeter) calculated at week 4. n = number of fish. One-way ANOVA (**A**, **B**) and Student's t-test (**C**–**F**): **p < 0.01; ****p < 0.0001. Error bars correspond to standard error of the mean (SEM). Scale bar: 7 mm.

In order to set up a fast and reliable DIO model in zebrafish, a 4-week overfeeding treatment was performed by providing dry food and freshly hatched artemia during the day (dry food: 15 mg (CTRL) vs. 52,5 mg (DIO)/fish/day; artemia: 6 mg (CTRL) vs 30 mg (DIO)/fish/day; see Suppl Fig. 1). The effects of such a diet were subsequently investigated for body weight, body length and BMI (Body Mass Index). After the first week of diet, a significant increase in body weight was observed and was maintained until week 4 in DIO fish compared to controls (CTRL) in both male and female groups (Fig. 1A,B). DIO-treated fish were markedly bigger than CTRL at week 4, corresponding to a 144.5% and 240% increase in body weight for males and females, respectively.

In addition, at the end of the experimental procedure, the body length of male and female fish was significantly higher in DIO-treated fish (108% and 122% increase in males and females, respectively) (Fig. 1C,D). The body mass index (BMI) was significantly increased in the DIO fish compared to CTRL (125% and 162% increase in males and females, respectively), suggesting that the gain in body weight is not only due to an increase in body length (Fig. 1E,F). Blood cholesterol and triglycerides levels were also measured and no significant changes were observed between CTRL and DIO fish (data not shown). Taken together, these data indicate that a 4-week overfeeding was sufficient to markedly increase body weight and BMI. Given that egg production could lead to a bias in body weight measurements in female and that sex hormones (i.e. estrogens) are known to impact brain plasticity including neurogenesis in both mammals and fish^34,35^, the next experiments were performed only in males.

**Figure 2:**
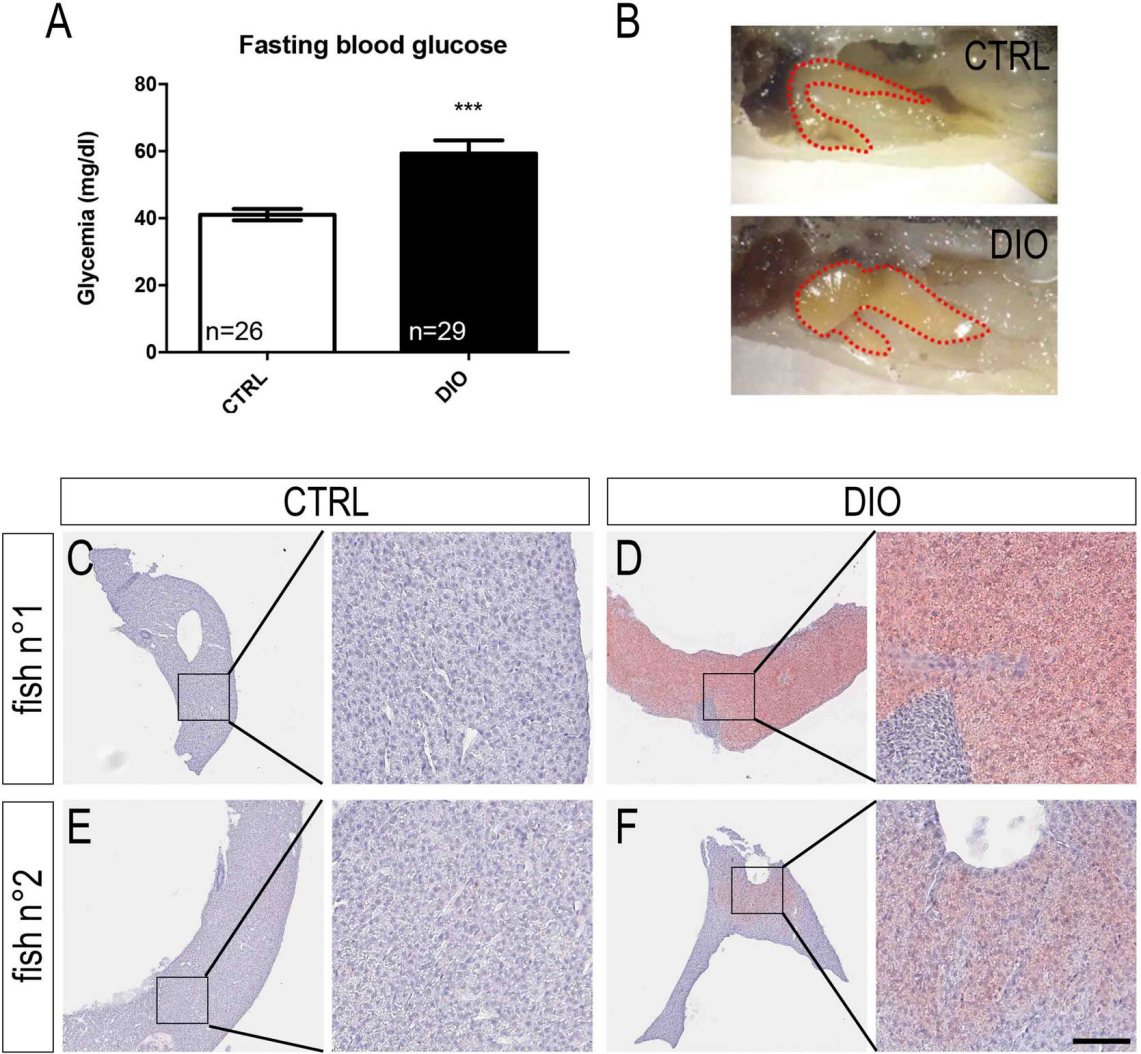
DIO leads to increased fasting blood glucose levels and to liver steatosis in zebrafish. (**A**) Fasting blood glucose measurements at week 4 in CTRL and DIO-treated fish. (**B**) Zebrafish liver pictures highlighting the increased size and yellowish color of liver in DIO-treated fish compared to CTRL. (**C**), (**E**) Liver sections stained with Oil Red O in two control fish showing no lipid accumulation. (**D**), (**F**) Liver sections stained with Oil Red O in two DIO-treated fish showing different levels of lipid accumulation (red color) and highlighting liver steatosis. Nuclei were stained with hematoxylin (**C**–**F**). These pictures are representative of 3 fish studied. n = number of fish. Student's t-test: ***p < 0.001. Error bars correspond to standard error of the mean (SEM). Scale bar: 1.5 mm (**A**); 250 μm for lower magnification pictures, 50 μm for higher magnification ones (**C**–**F**).

Numerous studies have previously shown that obesity and overweight result in metabolic disorders such as dysregulation in glucose and lipid metabolisms including the development of liver steatosis^5^. In the model developed in this study, fasting blood glucose was significantly increased in DIO-treated fish compared to CTRL (Fig. 2A). In addition, the liver from DIO fish was phenotypically bigger and more yellowish than those from CTRL, suggesting liver lipid accumulation (Fig. 2B). Oil Red O staining was consequently performed on liver cryo-sections to test this hypothesis. The liver of DIO fish exhibited an obvious red staining compared to controls (Fig. 2C–F), demonstrating hepatic lipid accumulation in overfed fish. Of note, the intensity of the liver red oil staining was heterogeneous among the DIO fish analyzed (Fig. 2D,F). Together, these data indicate that overfed fish exhibit dysregulations in glucose and hepatic lipid metabolism at week 4.

As a first conclusion, the phenotypic and metabolic data obtained from the DIO model strongly support that the developed protocol efficiently led to overweight and dysregulation in lipid and glucose metabolism.

### DIO induces BBB leakage, neuroinflammation and oxidative stress

Metabolic disorders such as diabetes and obesity are known to disrupt BBB^36,37^, an important interface corresponding to a highly selective barrier, that separates the blood flow from the fluids in the CNS. BBB disruption induced by obesity results in the leakage of substances into the brain and can consequently disrupt central homeostasis leading to increased brain inflammation and oxidative stress^37^. In the present study, DIO fish displayed an increased body weight and BMI and showed metabolism dysfunctions. Consequently, the impact of overfeeding was next investigated for brain homeostasis focusing on blood–brain barrier (BBB) physiology, neuroinflammation, oxidative stress and neurogenesis. Given the impact of sex steroids on brain homeostasis and peculiarly in zebrafish neurogenesis^38^, we decided to perform the following investigations in males.

**Figure 3:**
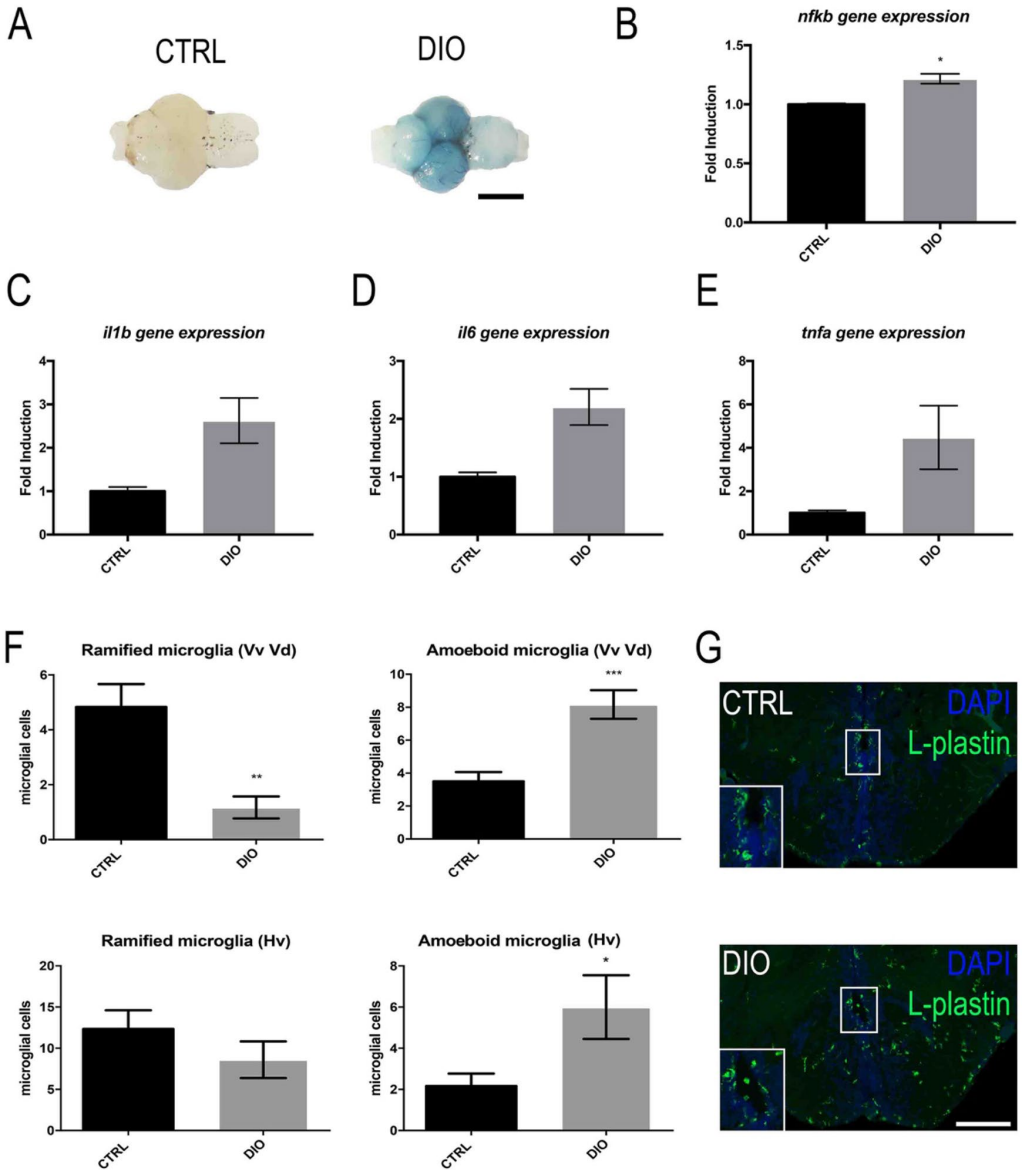
DIO increases BBB leakage and neuroinflammation. (**A**) Dorsal view pictures of the CTRL and DIO zebrafish brains following Evans blue staining. Note the blue staining observed in DIO-treated fish compared to controls (n = 6 brains). (**B**–**E**) qPCR gene expression analysis of *nfkb* and pro-inflammatory cytokines (*il1β, il6 and tnfα*) in CTRL and DIO fish (n = 3 pools of 2 brains). (**F**) Couting of ramified and amoeboid microglia in the ventral telencephalon (Vv Vd) and the anterior hypothalamus (Hv) regions in both CTRL and DIO-treated fish. (**G**) Representative L-plastin immunohistochemistry pictures showing microglia morphology in CTRL and DIO zebrafish brain in the subpallium (Vv Vd). Note the apparent increase in amoeboid-like morphology and the stronger L-plastin staining intensity in DIO fish compared to the control ones. n = number of fish. Student's t-test: * p < 0.05; **p < 0.01; ***p < 0.001. Error bar: standard error of the mean (SEM). Scale bar: 0.8 mm (**A**); 148 μm (**G**).

To investigate the impact of DIO on the BBB physiology, intraperitoneal injection of Evans Blue was performed, this dye quickly reaching the bloodstream. It results that overfeeding led to BBB leakage as revealed by the blue staining of the DIO brains (Fig. 3A; 5 brains out of 6 were blue) compared to CTRL, that mostly remained white (Fig. 3A; 5 brains out of 6 were white). The effect of overfeeding was next investigated on neuroinflammation by performing qPCR analyses. A consistent trend towards increased *il1*β, *il6* and *tnfα* gene expression was observed in the brains of DIO fish compared to those of controls (Fig. 3C–E). Furthermore, the pro-inflammatory *nfkb* transcription factor was slightly but significantly up-regulated (Fig. 3B). The morphology of microglial cells was also studied as a reflect of the cerebral neuroinflammatory state. In homeostatic conditions, microglial cells displayed a ramified morphology, while under activation, they became amoeboid (rounded morphology without ramifications) and exhibit phagocytic properties. By performing L-plastin immunohistochemistry, a switch in microglia morphology was observed (Fig. 3F,G). Microglial cells from DIO-treated fish appear more roundish and display stronger L-plastin staining along the neurogenic niches compared to controls (Fig. 3G). As shown in Fig. 3F, the number of ramified microglia was lower in DIO fish than in CTRL in the ventral telencephalon (Vv Vd: p-value = 0.0027) and in the anterior part of the hypothalamic region (Hv: p-value = 0.2762). In addition, the number of amoeboid microglial cells was significantly higher in DIO fish compared to controls in both regions (Fig. 3F). Taken together, these qPCR and IHC data demonstrate that DIO promotes neuroinflammation.

**Figure 4:**
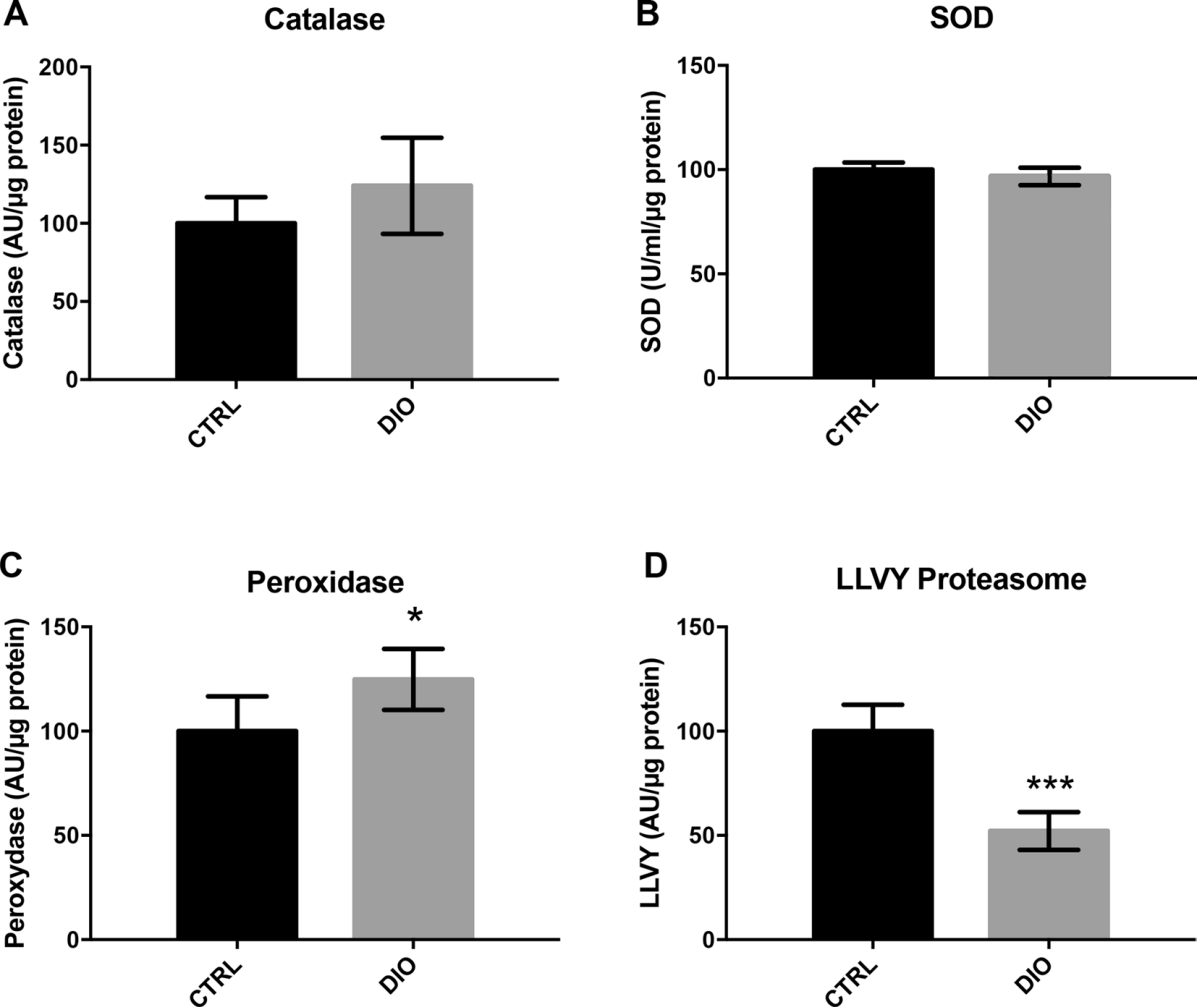
DIO disrupts antioxidant enzymes and proteasome activity in the brain of adult zebrafish. (**A**–**D**) Cerebral catalase, total superoxide dismutase (SOD), peroxidase and LLVY proteasome activities performed in lysates of adult zebrafish brain. n = 7 from two independent experiments. Student's t-test: *p < 0.05 ***p < 0.001. Error bar: standard error of the mean (SEM).

The dysregulations in lipid and glucose metabolisms observed in overfed fish could potentially affect redox homeostasis. The enzymatic antioxidant system associated with proteasome participates in the maintenance of this redox homeostasis. Consequently, some antioxidant enzymes activities (catalase, superoxide dismutase and peroxidase) and the chymotrypsin like activity of the proteasome were investigated in brain (Fig. 4). While there is no significant difference between DIO and control fish for SOD activity, a significant enhanced peroxidase activity was measured in brain lysates from DIO fish (+ 25%, p < 0.05 vs. CTRL) and a slight increase in catalase activity was also observed in DIO but failed to reach statistical significance (+ 24%, p = 0.09 vs. CTRL). These enhanced peroxidase and catalase activities could reflect the antioxidant response of the main detoxifying enzymes following a redox status imbalance in fish subjected to an overfeeding. In addition, a significant reduction of the chymotrypsin-like activity of the proteasome was measured in brain from DIO fish (− 48%, p < 0.001 vs. CTRL). This reduced proteasome activity may contribute to the altered redox status in overfed zebrafish leading to the accumulation of oxidized protein or lipid peroxidation products. In this line, we also demonstrated that the brain of DIO fish displayed stronger levels of 4-hydroxynonenal (4-HNE), a well-established end-product marker of lipid peroxidation (Fig. 8B).

Consequently, it appears that overfeeding disrupts brain homeostasis and promotes BBB leakage, cerebral inflammation and oxidative stress.

### DIO impairs adult neurogenesis and locomotor activity

**Figure 5:**
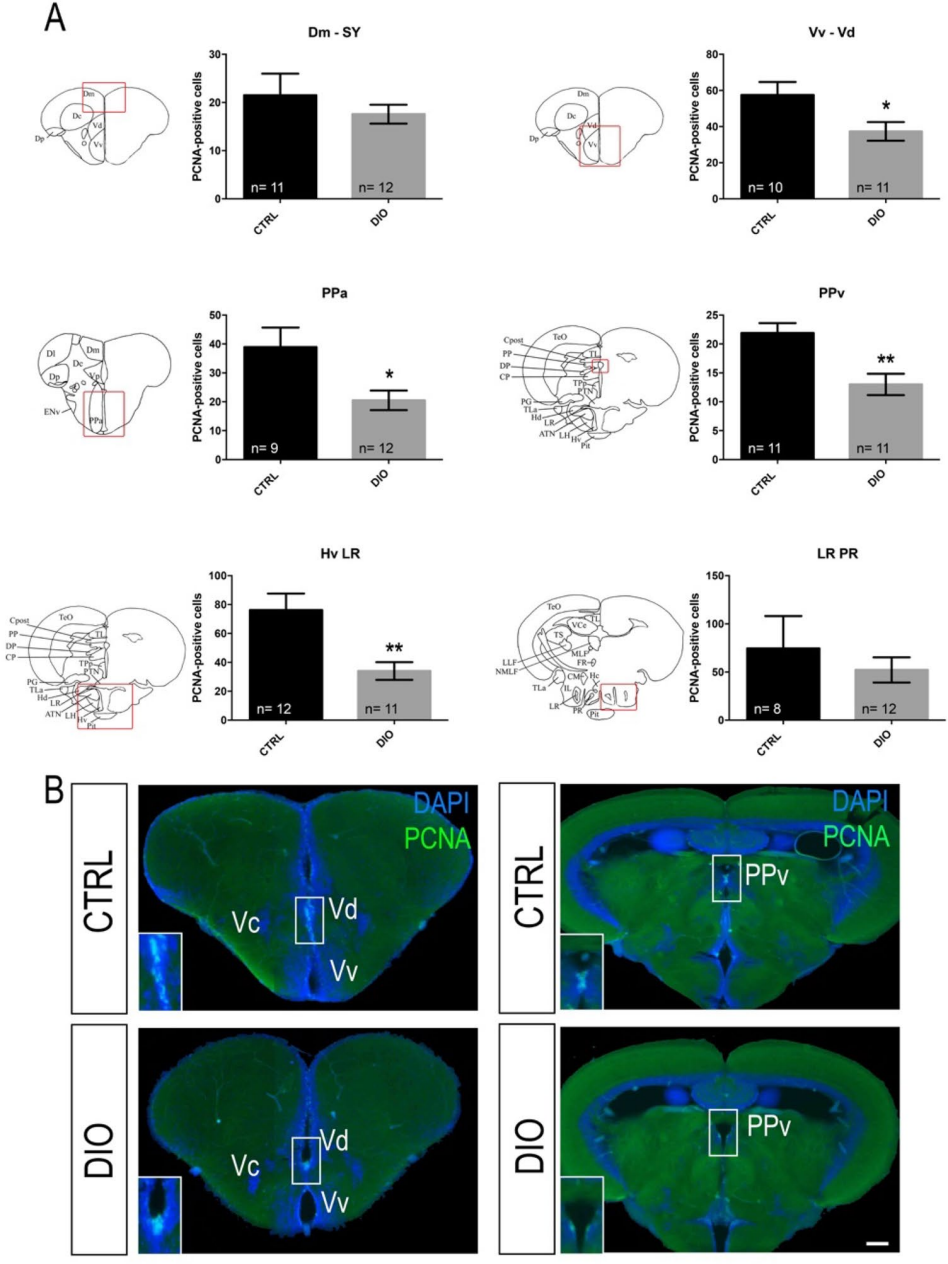
DIO impairs neurogenesis in the forebrain of adult zebrafish. (**A**) Statistical analysis of the number of proliferative cell (PCNA-positive) in CTRL and DIO-treated zebrafish. The respective brain schemes correspond to the transversal sections of the zebrafish brain for each studied region showing the main brain domains/nuclei according to the Zebrafish Brain Atlas from Wullimann et al. and were adapted from Menuet et al.^84,85^. A significant decrease in proliferative activity was observed between CTRL and DIO zebrafish in the Vv Vd, PPa, PPv and Hv LR neurogenic regions. (**B**) Representative digital pictures of PCNA immunohistochemistry (green) and cell nuclei counterstaining (DAPI in blue) on cryostat brain sections of CTRL (up) and DIO-treated fish (down). n = number of brains studied pooled from two independent experiments. Student's t-test: *p < 0.05 **p < 0.01. Error bar: standard error of the mean (SEM). Scale bar = 32 μm. Vv: ventral nucleus of ventral telencephalic area; Vd: dorsal nucleus of ventral telencephalic area; Dm: medial zone of dorsal telencephalic area; PPa: parvocellular preoptic nucleus, anterior part; PPv: periventricular pretectal nucleus; Hv: ventral zone of periventricular hypothalamus; LR: lateral recess of diencephalic nucleus; PR: posterior recess of diencephalic ventricle.

BBB disruption and neuroinflammation are well-established to be potential disruptors of adult neurogenesis. Brain cell proliferation in neurogenic niches was consequently studied by performing immunohistochemistry against the proliferative marker PCNA (Proliferating Cell Nuclear Antigen) in key neurogenic regions including the ventral (Vv-Vd) and dorsal (Dm) telencephalon, the anterior part of the preoptic area (Ppa), the periventricular pretectal nucleus (PPv) and two caudal hypothalamic regions (Hv LR and LR PR) (Fig. 5). Overfed fish displayed a general blunted neurogenesis in all the regions studied. Although, it did not reach statistical significance, a consistent trend was observed towards lower proliferation in the dorsomedian telencephalon (Dm SY) and around the lateral (LR) and posterior (PR) recess of the hypothalamic nucleus (Fig. 5A). Furthermore, a significant decrease in proliferative cells was observed along the neurogenic niches from the ventral (Vv) and dorsal (Vd) nuclei of the ventral telencephalic area, the anterior part of the parvocellular preoptic nucleus (PPa), the periventricular pretectal nucleus (PPv), the ventral zone of the periventricular hypothalamus as well as along the lateral recess of the diencephalic nucleus (Hv LR). Importantly, such decreased brain cell proliferation was observed in independent experiments, reinforcing the fact that overfeeding strongly resulted in blunted neurogenesis.

**Figure 6:**
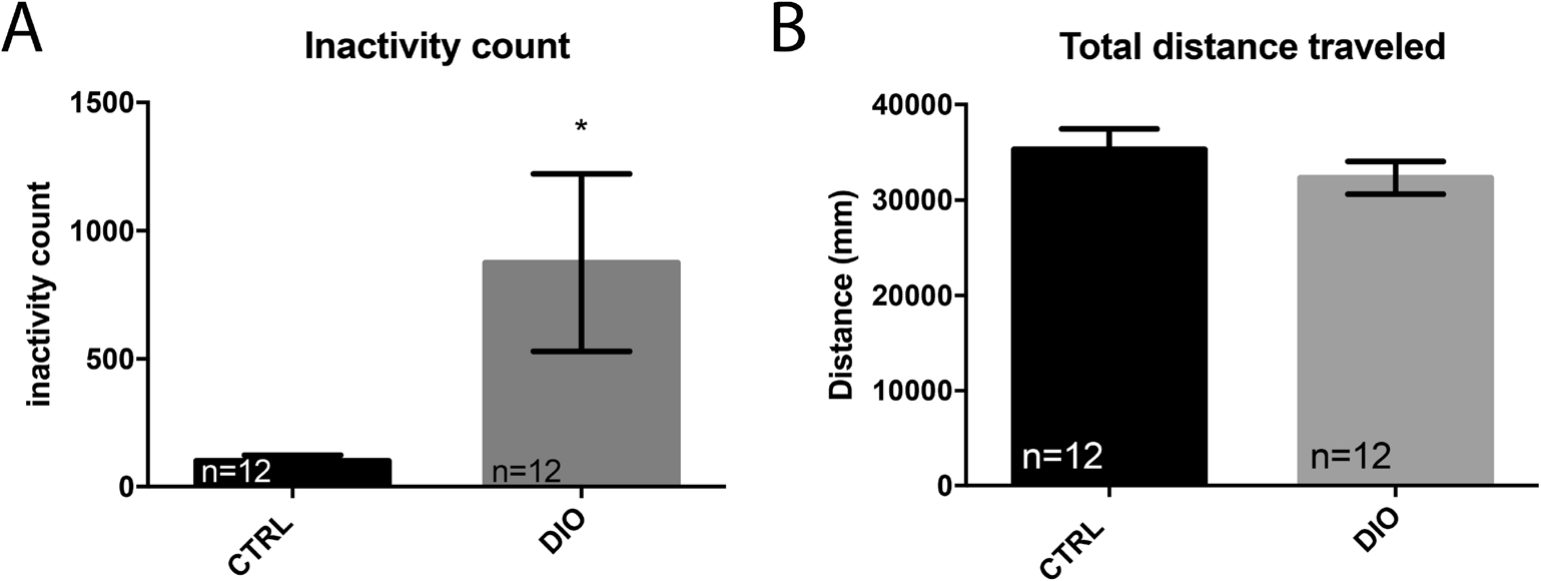
DIO increases the occurrence of inactivity in zebrafish. (**A**) graph showing the increased number of inactivity state (locomotion < 4 mm/sec) in DIO fish compared to controls, normalized to 100%. (**B**) graph showing that the total distance traveled during 10 min is similar between CTRL and DIO fish. n = 12 from 3 independent experiments. Student's t-test: *p < 0.05. Error bar: standard error of the mean (SEM).

We next addressed the potential effects of overfeeding on fish behavior and thus monitored the locomotor activity. By recording the locomotion of individual fish during a 10 min period, a significant increase in inactive state was observed in DIO fish compared to their respective controls, while the total distance traveled remained unchanged (Fig. 6). These data reveal the existence of different locomotion patterns between CTRL and DIO fish.

### *A. borbonica* herbal tea treatment prevents from deleterious effects induced by DIO

Overfed zebrafish appear as an appropriate model to test the effects of some compounds on weight gain and its complications^15,39,40^. *A. borbonica* was selected as an interesting candidate given its potential or demonstrated anti-oxidant, anti-inflammatory, and anti-diabetic according to its traditional use and to in vitro studies^29,32,33^.

First, the total phenolic acids content of the *A. borbonica* mother infusion (4 g/L), evaluated by using Folin-Ciocalteu assay, showed a concentration of 17.2 ± 0.9 mg GAE/g of plant dry powder. By using an aluminium chloride colorimetric method, the total flavonoids content determined was of 8.9 ± 0.5 mg EE/g of plant dry powder, twice less compared to phenolic acids content (Suppl. Figure 2). These results confirmed that *A. Borbonica* extract contains a substantial significant level of polyphenols and antioxidant properties as other medicinal plants as *Rosmarinus officinalis L* (16.67 ± 0.40 mg GAE/g)^41^. Then, a high-resolution mass spectrometry (HR-MS) analysis showed that a 4 g/L *A. Borbonica* infusion contained a variety of polyphenols including phenolic acids such as caffeic acid, caffeoylquinic acid, dicaffeoylquinic acid and some flavonoids such as Kaempferol hexoside and quercetin hexoside (Suppl. Figure 3). Caffeic acid derivatives including caffeoylquinic acid and dicaffeoylquinic acid were the most concentrated polyphenols identified in *A. Borbonica* infusion, at 1,278 ± 75 ng/mL and 531 ± 83 ng/mL, respectively.

**Figure 7:**
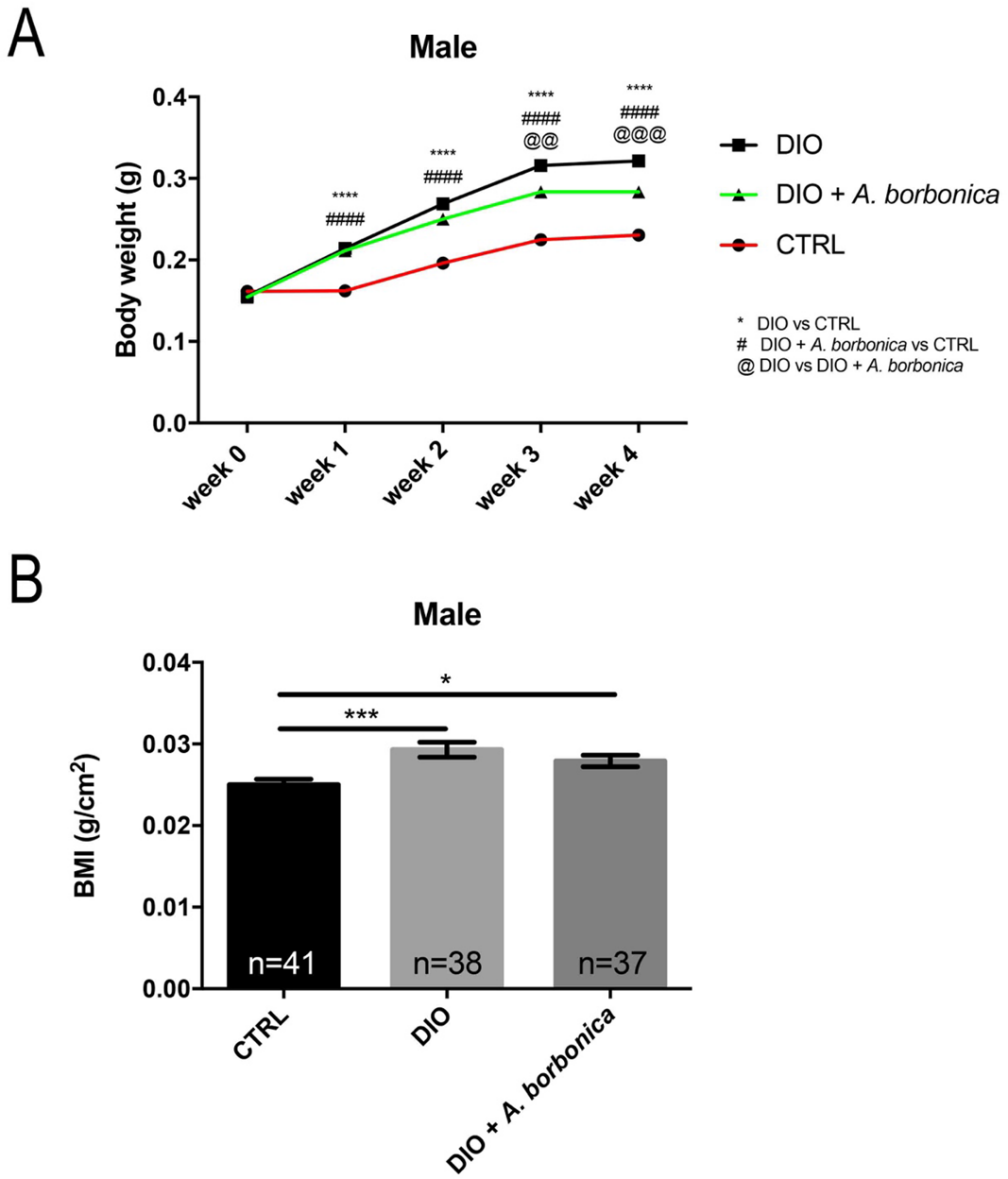
*A. borbonica* aqueous extracts slightly prevents from body weight gain and excessive BMI increase induced by DIO. (**A**) Line graph showing the increase in body weight of CTRL, DIO and DIO + *A. borbonica* during 4 weeks. (**B**) Graph showing the BMI of all groups at week 4**.** n = number of fish. One-way ANOVA (**A**) and Student's t-test (B): *p < 0.05 ***p < 0.001. Bar graph: standard error of the mean (SEM).

Next, to study the effects of *A. Borbonica* on weight gain and its consequences, DIO fish were treated overnight (6 pm to 8 am; 5 days a week) with water containing *A. borbonica* infusion (final concentration: 0.5 g/L water) during the 4-week period. We confirmed that overfed fish without plant extract displayed a significant increase in body weight from week 1 to week 4 compared to controls (Fig. 7A). Interestingly, DIO + *A. borbonica* did not result in any significant change in body weight during the first two weeks, compared to the DIO, but significantly exhibited a decrease in body weight gain during the third and fourth week compared to the DIO fish (Fig. 7A). At week 4 (Fig. 7B) DIO and DIO + *A. borbonica* fish displayed a significant BMI increase compared to CTRL but no significant difference was observed between DIO groups. The size of DIO and DIO + *A. borbonica* fish was also significantly higher compared to controls, but not significant differences were observed between the groups (data not shown). As well, *A. Borbonica* did not exhibit striking preventive effects on lipid deposits in the liver (data not shown) and did not strikingly prevent from increased fasting blood glucose levels induced by DIO (DIO vs CTRL: + 144.4%, DIO + *A. borbonica* vs CTRL: + 129%; n = 19–22, data not shown).

### *A. borbonica* aqueous extracts prevent BBB leakage and oxidative stress but has mitigated effects on neuroinflammation and neurogenesis

**Figure 8:**
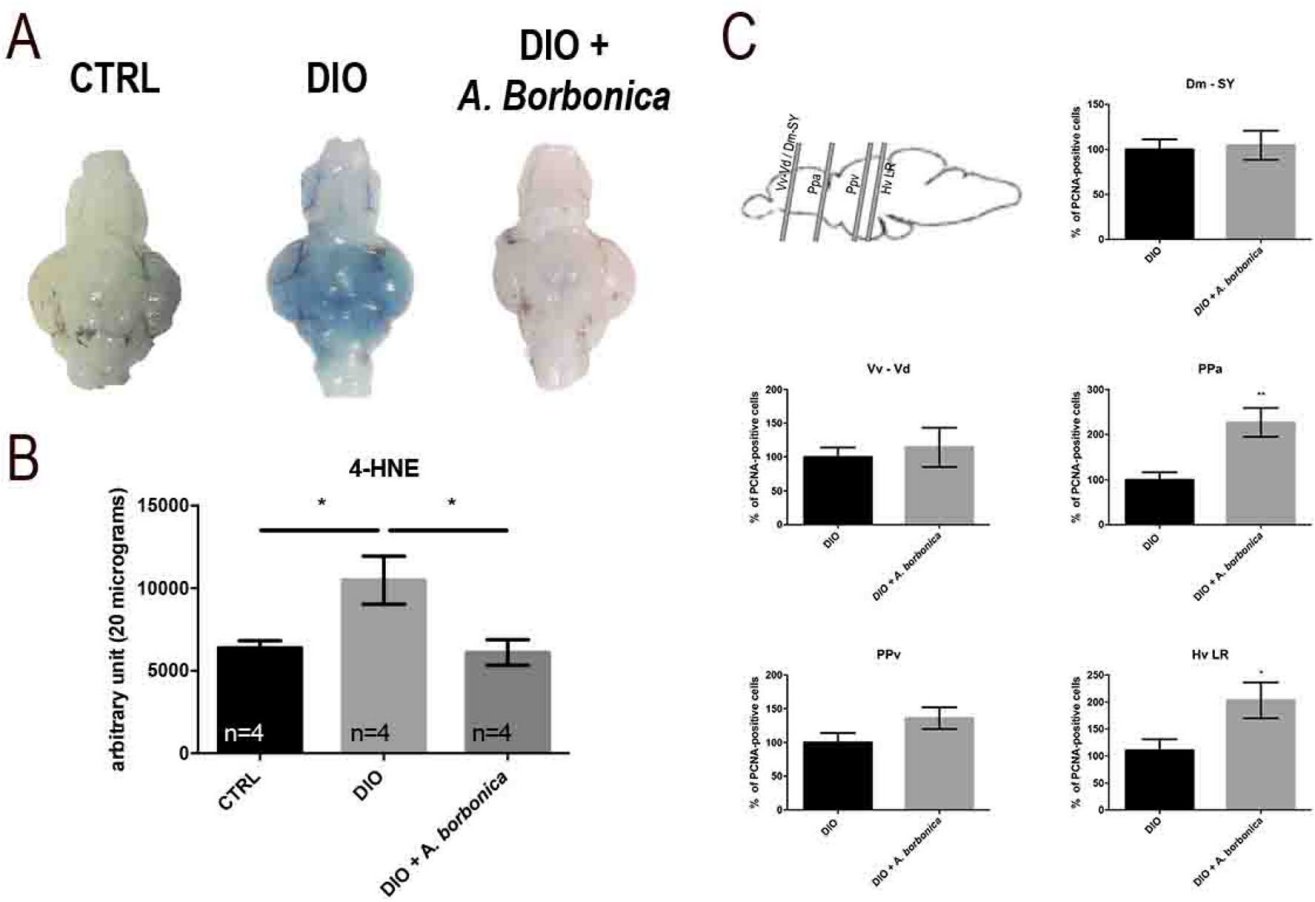
*A. borbonica* aqueous extracts treatment prevents from BBB leakage, brain oxidative stress and partially protects from the decreased neurogenesis induced by DIO. (**A**) Dorsal views of zebrafish brains after Evans Blue dye injection in CTRL, DIO, and DIO + *A. borbonica* treated fish. Zebrafish brains remain mostly white except for the DIO brains. (**B**) Dot blot quantification showing that the *A. borbonica* treatment in DIO fish prevents the cerebral increase in 4-HNE levels induced by overfeeding. n = number of brain fish studied. One-way ANOVA: *p < 0.05. Bar graph: standard error of the mean (SEM). (**C**) Statistical analysis of the number of proliferative cell (PCNA-positive) in DIO and DIO + *A. borbonica* treated zebrafish normalized to 100%. The sagittal brain scheme shows the corresponding section through the Dm-SY/Vv-Vd, the PPa, the PPv and the Hv LR. A weak significant increase in proliferative activity was observed between DIO and DIO + *A. borbonica* in the preoptic area (PPa) and the hypothalamic region (Hv LR). n = 8–11 fish. Student's t-test: *p < 0.05 **p < 0.01. Bar graph: standard error of the mean (SEM).

Given the anti-oxidant properties of *A. borbonica* infusion, its effect on the central disruptions (BBB leakage, oxidative stress and neurogenesis) induced by DIO was investigated. We confirmed that DIO resulted in BBB leakage and we showed that *A. Borbonica* extracts limited BBB disruption (Fig. 8A). Cerebral oxidative stress was next investigated by performing dot-blot against 4-hydroxynonenal (4-HNE), a well-established marker of oxidative stress. 4-HNE levels were increased in the brain of DIO-treated fish (Fig. 8B), and remained at basal levels in DIO + *A. Borbonica* treated fish showing that *A. borbonica* prevented the increase in oxidative stress induced by DIO (Fig. 8B). However, neuroinflammation in the brain of DIO + *A. Borbonica* appeared mitigated. Indeed, some pro-inflammatory cytokines were slightly decreased (i.e. *tnfa* fold induction is 1,36 *vs*. 4,47; data not shown) and microglial cells tended to be less amoeboid in the ventral telencephalon (Vv Vd) than in DIO fish, but remained activated in the hypothalamus (Hv), suggesting the persistence of a local neuroinflammatory state (data not shown).

Finally, the potential effect of *A. Borbonica* on impaired neurogenesis induced by overfeeding was also studied. It did not induce any striking change in brain cell proliferation in the neurogenic niches, except in the preoptic area and the caudal hypothalamus (Hv LR) for which proliferation was slightly up-regulated and was maintained at basal level (Fig. 8C). It suggests that *A. Borbonica* aqueous extract could partially rescue the impaired neurogenesis induced by overfeeding in a regional dependent manner.

## Discussion

We developed a reliable overfeeding zebrafish model resulting in many metabolic disorders including increased body weight, BMI and fasting blood glucose levels as well as the development of liver steatosis (Figs. 1, 2). Such increase in body weight and BMI appears more pronounced in female than male, probably in links with oogenesis and egg storage. In zebrafish, several DIO and/or HFD models have been proposed and lead to similar metabolic impairments as observed in our study, reinforcing the data obtained in this work^13–17,42,43^. The higher body weight and BMI observed in overfed fish should reflect the development of adipose tissue, as overfeeding protocols using artemia have been previously shown to promote subcutaneous and/or visceral adipocyte expansion in zebrafish^14,15,17^. In our work, we decided to use a mix of dry food and artemia in order to provide a diversity in the feeding as well as enrichment for zebrafish.

We also showed that DIO fish display heterogenous levels of liver steatosis. This pathological process was also previously observed in fish by Nakayama et al.^43^ in a 6-week DIO model using artemia. In contrast, Landgraf et al.^42^ observed hepatic steatosis only in fish overfed with both artemia and egg powder, but not with artemia alone, and this even after 8 weeks of treatment. Such differences could be explained by the quantity of food supplied to the overfed fish between these respective different protocols, and put our model as an intermediate one concerning liver steatosis.

Interestingly, overweight and obesity are known to be associated with insulin resistance and increased glycemia^44^. Lipid steatosis is also associated with fasting hyperglycemia and type 2 diabetes^45,46^. In our work, fasting blood glucose levels of DIO-treated fish were significantly higher than in controls (Fig. 2; ~ 40 mg/dl in CTRL vs ~ 60 mg/dl in DIO). Such an increase was previously obtained in fish overfed with artemia (without liver steatosis) but did not reach significant levels, while fish overfed with artemia and egg powder (with liver steatosis) exhibited a significant hyperglycemia^42^. Among the possible explanations for such differences, we could argue (1) for the power of the statistical analysis (26–29 fish/group in our study versus 10 for Landgraf et al*.*), (2) that the food quantity supplied in our study is higher than those from Landgraf’s one (137% increase in feeding), and (3) last but not least, it is tempting to speculate that the degree of liver steatosis could be correlated with hyperglycemia levels in accordance with the literature^47^, as it is known to promote chronic inflammation and insulin-resistance.

Taken together, these data highlight that the model developed in this study is relevant to other overfeeding models in the field. In addition, our data demonstrate that overfed zebrafish share common features with overweight/obese pathologies in human^1^. It consequently reinforces its use for studying the deleterious impact of overweight on several physiological processes such as brain homeostasis and plasticity.

Numerous works have shown that overweight and obesity were associated with cognitive impairments and that adult neurogenesis is involved in memory^11,48–50^, raising the question of the effects of DIO on brain homeostasis. In this context, the integrity of the blood–brain barrier and brain plasticity (i.e. neurogenesis) are two key parameters to investigate as they are linked to cognitive impairments. In our work, we report an increased BBB leakage, neuroinflammation and oxidative stress in the brain of overweight fish compared to controls (Figs. 3, 4, 8). These results are of peculiar interest given that oxidative stress and inflammation are known to promote BBB disruption^51,52^. The neuroinflammatory state observed in the brain of overfed fish through the up-regulation of pro-inflammatory cytokines and *nfkb* gene expression was reinforced by the switch from ramified to amoeboid microglia (Fig. 3). In a similar way, the impact of a high-glucose/high-cholesterol diet in zebrafish was recently associated with the increase in pro-inflammatory cytokines and *cd11b* gene expression, a microglial marker^53^, reinforcing the results obtained in our work. As well, these results should be also paralleled with the mammalian situation for which overweight and/or high fat diets in rodents induce neuroinflammation and microglial reactivity, mainly in the hypothalamus^54–56^.

Interestingly, in our experimental conditions, DIO fish display an increase in cerebral oxidative stress (Figs. 4, 8; increased 4-HNE levels and higher catalase and peroxidase activity), with a reduced proteasome activity that could contribute to impair redox balance in overfed fish and could result in increased oxidized protein or lipid peroxidation product accumulation. Indeed, oxidized proteins that are not degraded by the altered proteasome system may also contribute to the enhanced reactive oxygen species generation in DIO zebrafish tissues. For instance, increased 4-HNE accumulation observed in the brain of DIO fish may induce adduct formation in proteasome subunit leading to the inhibition of proteolytic activity. Very interestingly, it has been previously shown that oxidative stress homeostasis could affect the differentiation and proliferation of progenitor cells^57,58^, bringing evidence that redox imbalance, and generation of 4-HNE levels in the brain, could affect the behavior of the neural progenitor/stem cells.

Indeed, we demonstrated from independent experiments that DIO fish display a consistent and significant decrease in brain cell proliferation in most neurogenic niches including the ventral telencephalon, the preoptic area, the hypothalamus and the periventricular region of the pretectal nucleus (Fig. 5). It fits with another model of high caloric consumption in zebrafish resulting in decreased cerebellar proliferation^59^. In addition, in mammals, genetic and/or diet models of overweight/obesity were shown to result in decreased neural stem cell proliferation and subsequently to a lower number of newborn neurons generated^54,60–63^. Such a decreased neurogenesis was also observed in other metabolic disorders including hyperglycemia in both rodents and fish^18,64^. Further investigations would be required for determining the impact of overfeeding in newborn cell migration, differentiation and survival in both constitutive and regenerative conditions in zebrafish. As well, the identification of the misregulated extrinsic and intrinsic factors responsible for blunted neurogenesis should be further investigated, considering for example the Delta-Notch signaling pathway known for controlling NSC activity. In addition, in the offspring of mice that have followed a HFD, NSC upregulates Notch receptors and its downstream effector Hes, promoting NSC quiescence and limiting neurogenesis^65^. Also, given the links between neurogenesis and cognitive functions^50^, it is important to mention that a HFD zebrafish model was shown to display impaired cognitive functions as revealed by active avoidance test and by the disturbed expression of numerous genes involved in neuronal activity, anti-oxidative stress, and BBB functions^66^.

Consequently, we demonstrated for the first time in a same zebrafish model that overfeeding induces many detrimental effects on brain homeostasis as shown by BBB leakage, neuroinflammation, enhanced oxidative stress and impaired neurogenesis. These results also raised the question of the chicken or the egg between the first factors (inflammation? oxidative stress?) disrupting brain homeostasis and would allow to further investigate the mechanisms by which metabolic disorders disturb neurogenic activity and behavior.

*A. borbonica* herbal tea has been traditionally used as a natural remedy for its potential anti-inflammatory, anti-oxidant and anti-diabetic properties^29–33^. For this reason, we decided to work on aqueous extract. The subsequent HR-MS analysis allowed us to confirm previously published data demonstrating that *A. Borbonica* extracts contain high phenolic and flavonoid contents associated with antioxidant properties^29,33^. HR-MS and Folin-Ciocalteu assay analyses revealed that *A. Borbonica* infusion contains major phenolic acids including caffeic acids derivatives (dicaffeoylquinic acid and chlorogenic acid) and some glycosylated flavonoids (kaempferol hexosides and quercetin hexosides). In HFD mice, dicaffeoylquinic acids (150 mg/kg of body weight) improve metabolic parameters (i.e. liver and adipose tissue masses, decreased inflammatory factors, better hepatic lipid synthesis and degradation)^67^. A study of Jung et al.^68^ also showed the interesting effect of quercetin by regulating genes involved in lipid metabolism. As well, intraperitoneal injection of chlorogenic acid (100 mg/kg of body weight) was shown to ameliorate HFD-induced liver steatosis, insulin resistance and adipocyte hypertrophy in mice^69^. In addition, caffeic acids including dicaffeoylquinic acid and chlorogenic acid have been shown to exert antioxidant properties especially in the brain^70–72^. Although *A. borbonica* did not prevent from liver steatosis in our experimental conditions, and did not significantly decrease fasting blood glucose levels, a decreasing trend was observed (DIO vs CTRL: + 144.4% increase in fasting glycemia while DIO + *A. borbonica* vs CTRL: + 129% increase in fasting glycemia). Importantly, *A. borbonica* treatment significantly limited weight gain from week 3 to week 4 compared to DIO, without modulating feeding behavior (data not shown). This result is of peculiar interest given that the BMI of DIO + *A. borbonica* fish is higher than CTRL, but this increase is less significant than the one of DIO vs. CTRL (Fig. 7), while the size of DIO and DIO + *A. borbonica* remain unchanged (data not shown: 33.46 mm for DIO vs 33.04 mm for DIO + *A. borbonica*-). This result have to be paralleled with those in HFD mice for which treatment with chlorogenic acid and dicaffeoylquinic acid avoid or limited gain weight^67,69^. The effects observed in our study are probably less spectacular given the low concentrations of these polyphenols.

In addition, DIO + *A. Borbonica* fish are protected from BBB leakage and brain oxidative stress induced by DIO (Figs. 7, 8). These preventive effects could be attributed *A. Borbonica* anti-oxidant activity supported by its polyphenol content (phenolic acids, flavonoids and tannins) and are consistent with in vitro studies showing its anti-oxidant properties^29,33^. Interestingly, in almost all the neurogenic niches studied, DIO fish treated with *A. borbonica* still display blunted neurogenesis that nevertheless appeared less severe in the preoptic area and the hypothalamus. Consequently, *A. borbonica* did not fully prevent from neurogenic defects induced by DIO, showing that other mechanisms than BBB leakage and oxidative stress should be involved in such neurogenic impairments. It would be also interesting to determine the *A. borbonica* metabolites found within the brain fish in order to elucidate which compounds could be responsible for the central protective effects.

## Conclusion

To conclude, we have developed an overfeeding model in zebrafish that mimics the mammalian overweight state in the periphery and also in the central nervous system. To our knowledge, this is the first report showing the deleterious impact of DIO on brain homeostasis and especially considering the forebrain neurogenesis in zebrafish.

*A. borbonica* aqueous extract (mimicking herbal tea consumption) was shown to limit significantly weight gain in overfed zebrafish. The DIO protocol developed in this work could serve as a new physiological screening tool for identifying “anti-overweight” and “anti-obesity” drugs. It will allow the discovery of new preventive and therapeutic treatments against weight gain and associated central deleterious effects such as BBB leakage, neuroinflammation, oxidative stress and impaired neurogenesis. Although *A. borbonica* aqueous extract has only a limited effect on body weight and associated BMI, it prevents BBB leakage, cerebral oxidative stress and partly improve neurogenesis. Thus, natural compounds have a limited effect on the body weight but could prevent some central disorders induced by overfeeding.

## Material and methods

### Animals and ethics

Three to four month-old adult wildtype male and female zebrafish (*Danio rerio*) were obtained from our zebrafish facility and were maintained under standard conditions of temperature (28.5 °C), photoperiod (14 h dark/10 h light), pH (7.4) and conductivity (400 μS). The gender of the animal was performed visually according to sexual dimorphisms (males are thinner and females have a bigger belly). All experiments were conducted in accordance with the French and European Community Guidelines for the Use of Animals in Research (86/609/EEC and 2010/63/EU) and approved by the local Ethics Committee for animal experimentation of CYROI and the French Government (APAFIS_ 20191105105351_v10).

### Diet-induced overweight/obesity (DIO) protocol

Adult zebrafish (3–4 months) were divided into 2 or 3 groups (control and DIO or CTRL, DIO, DIO + *A. borbonica*) at the same density between each group (10 to 20 fish maximum per 3.5 L tank, according to the experiment); males and females being mixed in each tank. The control group was fed once a day with dry food in the morning (15 mg/fish/day, GEMMA 300, Planktovie) and freshly hatched artemia (6 mg/fish/day, Artemia cysts; REF: B052-P) in the afternoon. The DIO and DIO + *A. Borbonica* group was fed six times a day with dry food (52.5 mg/fish/day) and three times with freshly hatched artemia (30 mg/fish/day) in the afternoon. These treatments were performed on a four-week period (see Suppl Fig. 1).

### *A. Borbonica* herbal tea treatments

Leaves of *A. borbonica* (Bois d’osto; Saint-Joseph de La Réunion; REF: BOSJDTCA171218AA) were obtained from the Cooperative des Huilles Essentielles de Bourbon (CAHEB). Leaves were crushed and stored at − 20 °C. Infusions were prepared every day by mixing 1 g of crushed plants with 250 mL of boiled fish water for 10 min. After filtering, the 250 mL of *A. borbonica* infusion were added to 1,750 mL of fish water to reach a final volume of 2L and a final *A. borbonica* concentration of 0.5 g/L. The DIO fish treated with *A. borbonica* were fed as the DIO fish during the day from 8 AM to 6 PM and treated with the plants from 6 PM to 8 AM (5 days a week). During the infusion treatment, all the groups (CTRL, DIO, DIO + *A. borbonica*) were maintained out of the system.

### Body weight, body mass index (BMI) and fasting blood glucose measurements

Fish of each group were weighted every week. They were captured using a net, quickly dried on a tissue paper, briefly weighed and immediately placed back into water. This procedure is usually done in less than 20 s. In addition, the body length of the fish was measured at the beginning (first day, during the weighing process) and at the end of the experiment (week 4, after euthanasia—see below-) from the tip of the mouth to the end of the tail (total length). The body mass index (BMI) was calculated by dividing the body weight (g) with the square of the length (cm^2^).

For blood glucose measurements, fish were fasted the day before and euthanized using ice water (2–4 °C) in order to avoid blood glucose fluctuations due tricaine use^73^. Fish were gently dried with a tissue and one eye was removed allowing the ocular cavity to fill with blood. The glycemia (mg/dl) was measured using a glucometer (One-Touch Ultra, LifeScan, France), as previously described^19^.

### Blood collection for metabolic analyses

The fish were first euthanatized with tricaine, then one eye was removed and the blood was collected using a pipet that was previously equilibrated by 1X PBS and EDTA (1 mL of 1X PBS  is added in EDTA blood tube and vortexed). The blood of 5 fish was pooled (around 10 µL) in one Eppendorf with 40 µL of 1X PBS 1 × EDTA to avoid its clotting. After that, the blood samples are centrifuged and the plasma was collected frozen at − 80 °C.

### Cholesterol and triglyceride measurements

The blood of 5 fish (around 10 µL) was collected and pooled in one tube containing 40 µL of 1X PBS EDTA. 20 µL of plasma in the tube is diluted to the half with 1X PBS EDTA. 1 µL of diluted plasma is plotted in 96 well plate and cholesterol measurements were performed using the kit of Cholesterol FS according to manufacturer’s recommendation (DiaSys Diagnostic Systems—CODE CQN: KS from DiaSys). For triglyceride measurement, Triglyceride FS kit was used (DiaSys Diagnostic Systems—CODE CQN: KS from DiaSys). The absorbance at 500 nm was then measured using TECAN SUNRISE and cholesterol and triglyceride concentrations were determined.

### Tissue preparation

At the end of the experimental period, fish were euthanatized before being fixed in 4% PFA (Paraformaldehyde) dissolved in 1X Phosphate Buffer Saline (PBS).

For cryostat sections, the brain and the liver were dissected and cryopreserved by an overnight incubation in 1X PBS, containing 30% sucrose. Then, they were embedded in OCT matrix and cut using a cryostat at 12 µm thickness.

For qPCR or protein analyses, the tissues of interest were immediately dissected, snap-frozen and kept at − 80 °C.

### Immunostaining

For immunohistochemistry experiments, cryostat sections were rehydrated twice with 1X PBS containing 0.2% Triton (PBS-T). Antigen retrieval was performed using sodium citrate (pH 6) heated at 80 °C for 15 min. Sections were washed twice in PBS-T before being blocked in PBS-T containing 2% BSA. Next, sections were incubated with the following primary antibodies: rabbit anti zebrafish L-plastin (kindly provided by Dr Michael Redd^74^) and/or mouse anti-PCNA (1:100; clone PC10, Dako; RRID: M0879) overnight at room temperature. The slides were then washed twice in PBS-T and incubated with DAPI (4′,6′-diamidino-2-phenylindole) and secondary antibodies: donkey anti-rabbit Alexa Fluor 488 for L-plastin (1:300; REF: A21206; Life Technologies, Bethesda, MD; RRID: AB_10049650) and goat anti-mouse Alexa Fluor 594 or 488 for PCNA (1:300; REF: A11005 for Alexa 594 and A28175 for Alexa 488; Life Technologies; RRID: AB_141372 and AB_2536161) for 1h30 at room temperature. Sections were rinsed and the slides were mounted with anti-fading medium (IMM Ibidi; REF: 50001). Note that PCNA antibody allows the detection of proliferative cells along the neurogenic niches as previously described^75^, and L-Plastin antibody the detection of microglial cells, displaying ramified processes when quiescent, or amoeboid morphology when activated (phagocytic functions)^76^.

### Oil Red O staining

Frozen liver sections were dried, rehydrated and rinsed with 60% isopropanol before being stained for 40 min with the freshly prepared Oil Red O (Sigma-Aldrich; REF: 00625) according to standard protocol. Nuclei counterstaining was performed by incubating the sections for 30 s with Mayer’s hematoxylin. Finally, the sections were dehydrated and mounted with mounting medium (IMM Ibidi; REF: 50001).

### Investigation of BBB permeability (Evans Blue dye injection)

For investigating BBB physiology, Evans Blue dye was used as a tracer to monitor BBB permeability^77^. Briefly, fish were anesthetized with 0.02% tricaine and intraperitoneally injected with freshly prepared 1% Evans Blue dye diluted in 1X PBS (10 µL of 1% Evans Blue for 0.1 g of zebrafish body weight). Fish were allowed to recover for 10 min before being sacrificed. Then, fish heads were fixed with 4% PFA-PBS. After, the brains were dissected and imaged.

### Protein extraction and dot blot

Zebrafish brain were lysed with Tris HCL buffer (50 mM pH7.4 EDTA 0.01 mM) and centrifuged (10,000 rpm, 4 °C for 5 min). Supernatants were kept at − 80 °C. Protein concentration was determined according to Bradford protein assay following manufacturer’s protocol. For dot blot, 20 µg of protein were plotted on a nitrocellulose membrane. Following Ponceau S (Ponceau Red) staining, membranes were blocked in blocking buffer (5% milk in 1 × PBS containing 0.02% Tween 20) and subsequently incubated for 1h30 with rabbit anti-4-hydroxynonenal (4-HNE, a marker of oxidative stress) antibodies (Abcam; REF: ab46545). Next, membranes were washed and incubated with secondary antibody coupled to HRP (1:2000) (Jackson’s laboratories; Goat anti-Rabbit coupled with HRP, REF: JII035003) for 1h30, before being revealed with enhanced chemiluminescence substrates and imaged with Amersham Imager 680.

### Protein extraction for cerebral antioxidant activities

For enzymatic activities determination, protein isolation from brains was performed as follow: between 4 to 8 mg of zebrafish brain previously collected and stored at − 80 °C were homogenized with a TissueLyser II (Qiagen) in 100 µL of Tris buffer (Tris (25 mM), EDTA (1 mM), pH 7.4). After centrifugation (5,000 rpm, 4 °C for 10 min), the supernatant was used for protein quantification and enzymatic assays. Total protein concentration of lysates was quantified by the bicinchoninic acid assay (BCA).

The catalase activity assay was estimated on 15–20 µg of protein lysates in 25 mM Tris–HCl (pH 7.5), using protocols previously described^78,79^. Blanks were measured at 240 nm just before adding 80 µL of H_2_O_2_ (10 mM final) to start the reaction. Catalase activity was determined by measuring the absorbance at 240 nm and was calculated using a calibration standard curve of increasing amount of catalase between 12.5 and 125 units/mL. Catalase activity was expressed as international catalytic units per mg of proteins and then normalized in percentage versus the control condition.

Total SOD activity was determined using the cytochrome *c* reduction assay, as previously described^80^. In this method, superoxide radicals generated by the xanthine/xanthine oxidase system reduce cytochrome *c*, thereby leading to an increase in absorbance at 560 nm. 20 µL aliquot (about 10 µg of protein) of the lysates was combined with 170 µL reaction mixture (xanthine oxidase, xanthine (0.5 mM), cytochrome *c* (0.2 mM), KH_2_PO_4_ (50 mM, pH 7.8), EDTA (2 mM) and NaCN (1 mM)). The reaction was monitored in a microplate reader (Fluostar OPTIMA, BMG Labtech France) at 560 nm for 1 min, at 25 °C. Total SOD activity was calculated using a calibration standard curve of SOD (up to 6 units/mg). Results were expressed as international catalytic units per milligram of cell proteins and then normalized in percentage versus the control condition.

Peroxidase activity of tissue lysates was assessed according to the protocol described by Everse and colleagues^81^. A reaction mixture was prepared with 200 µL of 50 mM citrate buffer/0.2% o-dianisidine and 20 µL of lysates (between 5 to 10 µg of protein). The reaction was initiated by adding 20 µL of 200 mM H_2_O_2_. Peroxidase activity was determined by measuring the absorbance at 450 nm at 25 °C for 3 min. Peroxidase activity was expressed as international catalytic units per mg of proteins and then normalized in percentage versus the control condition.

Chymotrypsin-like activity of the proteasome was assayed using fluorogenic peptide (Sigma-Aldrich, St Louis): Suc-Leu-Leu-Val-Tyr-7-amido-4-methylcoumarin (LLVYMCA at 25 mM), as described previously^78^. Analyses were carried out with 5–10 μg of protein in 25 mM potassium phosphate buffer (pH 7.5) containing LLVY-MCA, at 37 °C for a 0–30 min incubation period. The fluorescence of aminomethylcoumarin was determined at excitation/emission wavelengths of 350/440 nm using a microplate spectrofluorometer reader (Fluostar OPTIMA, BMG labtech France). Peptidase activity was measured in the absence or in the presence of 20 µM proteasome inhibitor MG132 (N-Cbz-Leu-Leu-leucinal) and the specific proteasome activity was obtained by subtracting the residual activity (not inhibited by MG132), i.e. total cellular peptidase activity and non-proteasomal peptidase activity.

### RNA extraction and reverse transcription

Zebrafish brains were removed from the skull, pooled (n = 2), and stored at − 80 °C prior to RNA extraction. Three pools of 2 brains from control and DIO-treated fish were grinded with TissueLyser II (Qiagen, Chatsworth, CA) and RNA extraction was performed using RNA easy Mini Kit (Qiagen) according to manufacturer’s protocol. Then, 2 µg of RNA were reverse transcribed into cDNA using random hexamer primers (Invitrogen, REF: 100026484) and MMLV reverse transcriptase (Invitrogen, REF: 28025-021).

### Gene expression analysis by qPCR

Semi-quantitative PCR experiments were performed using the Biorad CFX Connect Real-Time System (BR006305) using the SYBR green master-mix (Eurogentec) and specific zebrafish primers. Each PCR cycle was conducted for 15 s at 95 °C and 1 min at 60 °C. Melting curve analyses and PCR efficiency were performed to confirm correct amplification. Results were analyzed and the relative expressions of the pro-inflammatory cytokine genes (*il1*β*, il6 and tnfα*) and *nfkb* were normalized against the housekeeping *ef1a* gene. The sequences of the primers are provided in Table 1.

**Table 1:** Zebrafish qPCR primer sequences of *ef1α, il8, ilβ, tnfα and il6.*

Gene	Forward primer	Reverse primer
*ef1α*	AGCAGCAGCTGAGGAGTGAT	CCGCATTTGTAGATCAGATGG
*Il1β*	GCTGGAGATCCAAACGGATA	ATACGCGGTGCTGATAAACC
*tnfα*	GCGCTTTTCTGAATCCTACGT	GCCCAGTCTGTCTCCTTCT
*il6*	TCAACTTCTCCAGCGTGATG	TCTTTCCCTCTTTTCCTCCTG
*nfkb*	CGGCCCACTGTAGTTGTG	TGCGTTTCCGTTATAAGTGTG

### Microscopy

Micrographs were obtained with an Eclipse 80i Nikon microscope equipped with a Hamamatsu digital camera (Life Sciences, Japan), and with a nanozoomer S60 (Hamamatsu). Pictures were adjusted for brightness and contrast in Adobe Photoshop.

### Cell counting

For analyzing constitutive neurogenesis, proliferative activity was determined by quantification of PCNA-positive from 2 to 3 cryostat consecutive sections (12 µm thickness/section) by region of interest. Images were analyzed for detection of PCNA positive nuclei using ImageJ software (National Institutes of Health, Bethesda, MD; RRID: SCR_003070) by adjusting parameters (threshold, binary, and watershed). Briefly, the parameters were set up as follows for each picture: threshold 65, 255, particle size 200-infinity. Minor modifications in these parameters could be slightly adjusted according to the experiments. In addition, ImageJ automated selection of PCNA-positive nuclei was manually double-checked and adjusted if necessary, for each picture. Neuroanatomical structures were identified with DAPI counterstaining. Cell counting was performed in blind conditions by two different people and the provided graphs correspond to the mean of proliferative cells per section.

For determining the number of resting and amoeboid microglia, manual counting was performed on 2 consecutive brain cryosection (12 µm thickness/section) for each region of interest (ventral telencephalon and anterior hypothalamus). The counting was performed on a total of 5 to 6 fish per condition, and the provided graphs correspond to the mean of proliferative cells per section.

### Behavioral analysis

The locomotor activity of zebrafish was monitored by the ZebraCube equipment (Viewpoint). Fish were placed in tanks within the ZebraCube equipment. Locomotor activity was recorded using the viewpoint software and inactivity, small and large activity counts, distance and duration were analyzed. A total number of 12 fish (from 3 independent experiments) were subjected to behavioral analysis. Individual fish were placed in separate tanks in an equal volume of water (750 mL in each tank, corresponding to a column height of 7 cm). The tanks were placed in the ZebraCube equipment with equivalent distance between them and a separation was placed between the tanks in order to avoid visual interaction between the fish from CTRL and DIO groups. The fish were allowed to discover freely this new environment for 10 min in order to adapt to the new space without excessive amount of stress. Then, the locomotor activity was recorded for 10 min: the movement of the fish was tracked as follows: the inactivity (< 4 mm/s), small activity (4–8 mm/s) or high activity (> 8 mm/s).

### Instrumentation and LC–MS/MS Conditions

Polyphenols extracted from *A. borbonica* infusion were identified by Ultra-high-performance liquid chromatography coupled with diode array detection and HESI-Orbitrap mass spectrometer (Q Exactive Plus, Thermo Fisher). Briefly, 10 µL of sample was injected using an UHPLC system equipped with a Thermo Fisher Ultimate 3,000 series WPS-3000 RS autosampler and then separated on a C18 column (5 µm, 4.6 mm × 100 mm, Thermo Fisher Scientific Inc.). The column was eluted with a gradient mixture of 0.1% formic acid in water (A) and 0.1% formic acid in acetonitrile (B) at the flow rate of 0.450 mL/min, with 5% B at 0.00 to 0.1 min, 75% B at 0.1 to 7.1 min, 95% B at 7.2 to 7.9 min and 5% B at 8.0 to 10 min. The column temperature was held at 30 °C and the detection wavelengths were set to 280 nm and 310 nm.

For the mass spectrometer conditions, a Heated Electrospray Ionization source II (HESI II) was used. Nitrogen was used as drying gas. The mass spectrometric conditions were optimized as follows: spray voltage = 2.8 kV, capillary temperature = 350 °C, sheath gas flow rate = 60 units, aux gas flow rate = 20 units and S lens RF level = 50.

Mass spectra were registered in full scan mode from m/z 100 to 1,500 in negative ion mode at a resolving power of 70,000 FWHM at m/z 400. The automatic gain control (AGC) was set at 1e^6^. The Orbitrap performance in negative ionization mode was evaluated weekly and external calibration of the mass spectrometer was performed with a LTQ ESI negative ion calibration solution (PIERCE). Identification of the compounds of interest was based on their exact mass, retention time and MS/MS analysis. Data were acquired and processed by XCalibur 4.0 software (Thermo Fisher Scientific Inc.).

### Identification and quantification of polyphenols in *Antirhea borbonica* infusion

To determine total phenolic acids content in plant extract, Folin-Ciocalteu test was used^82^. Briefly, in a 96-well microplate, 25 μL of plant extract, 125 μL of Folin-Ciocalteu’s phenol reagent (Sigma Aldrich) and 100 μL of sodium carbonate (Sigma Aldrich) were added and incubated at 50 °C for 5 min and then at 4 °C for 5 min. The absorbance was measured at 760 nm (FLUOstar Optima, BMG Labtech). A calibration curve between 12.5 − 300 µM was prepared using a standard solution of gallic acid (Sigma-Aldrich, Germany). Total phenol content was expressed as g gallic acid equivalent (GAE) per g plant powder.

Total flavonoids content was also measured using the aluminium chloride (AlCl_3_) colorimetric assay and adapted from Zhishen et al.^83^. For this measurement, 100 μL of herbal tea extract were mixed in a 96-well microplate with 6 μL of 5% aqueous sodium nitrite (NaNO_2_) solution. After 5 min, 6 μL of 10% aqueous AlCl_3_ were added and the mixture was vortexed. Then, after 1 min incubation, 40 μL of 1 M NaOH were added. The absorbance was read at 510 nm (FLUOstar Optima, BMG Labtech). A calibration curve between 6.25–300 µM was prepared using a standard solution of epicatechin (Sigma-Aldrich). Total flavonoid content was expressed as g epicatechin acid equivalent (EE) per g plant powder.

The identification and the quantification analysis of caffeic acid, dicaffeoylquinic acid, caffeoylquinic acid, quercetin and kaempferol were achieved by an LC–MS/MS analysis as previously described by^29^, with minor modifications. For the mass spectrometry quantification, a mixed stock solution containing 10 µg/mL of each polyphenols was prepared in methanol.

Calibration curves were constructed by plotting the peak area of the analytes *versus* the concentration of the analytes with linear regression using standard samples at eleven concentrations. The calibration curves of each polyphenols had a correlation coefficient (R^2^) of 0.99.

### Statistical analysis

Comparisons between two groups were performed using a statistical Student’s t-test. If more than two groups were analyzed, multiple testing was performed by one-way ANOVA. Error bars correspond to the standard error of the mean (SEM), and n values correspond to the number of animals or to the number of samples for all experiments. P-values < 0.05 were considered statistically significant (*p < 0.05, **p < 0.01, ***p < 0.001, ****p < 0.0001).

## Supplementary information

Supplementary Information 1.
