# Supplementary material for: Impaired brain homeostasis and neurogenesis in diet-induced overweight zebrafish: a preventive role from A. borbonica extract
Source: Sci Rep. 2020 Sep 2;10:14496. doi: 10.1038/s41598-020-71402-2 (PMC7468118; doi:10.1038/s41598-020-71402-2)
Supplement: Supplementary file 1 — Supplementary Information 1. [file 41598_2020_71402_MOESM1_ESM.docx]

**Impaired brain homeostasis and neurogenesis in diet-induced overweight zebrafish:**

**A preventive role from *A. borbonica* extract**

Batoul Ghaddar^1^, Bryan Veeren^1^, Philippe Rondeau^1^, Matthieu Bringart^1^, Christian Lefebvre d’Hellencourt^1^, Olivier Meilhac^1,2^, Jean-Loup Bascands^1^ and Nicolas Diotel^1*^

**^1^** Université de La Réunion, INSERM, UMR 1188, Diabète athérothrombose Thérapies Réunion Océan Indien (DéTROI), Saint-Denis de La Réunion, France

**^2^** CHU de La Réunion, Saint-Denis, France

* corresponding author: [nicolas.diotel@univ-reunion.fr](mailto:nicolas.diotel@univ-reunion.fr)

**
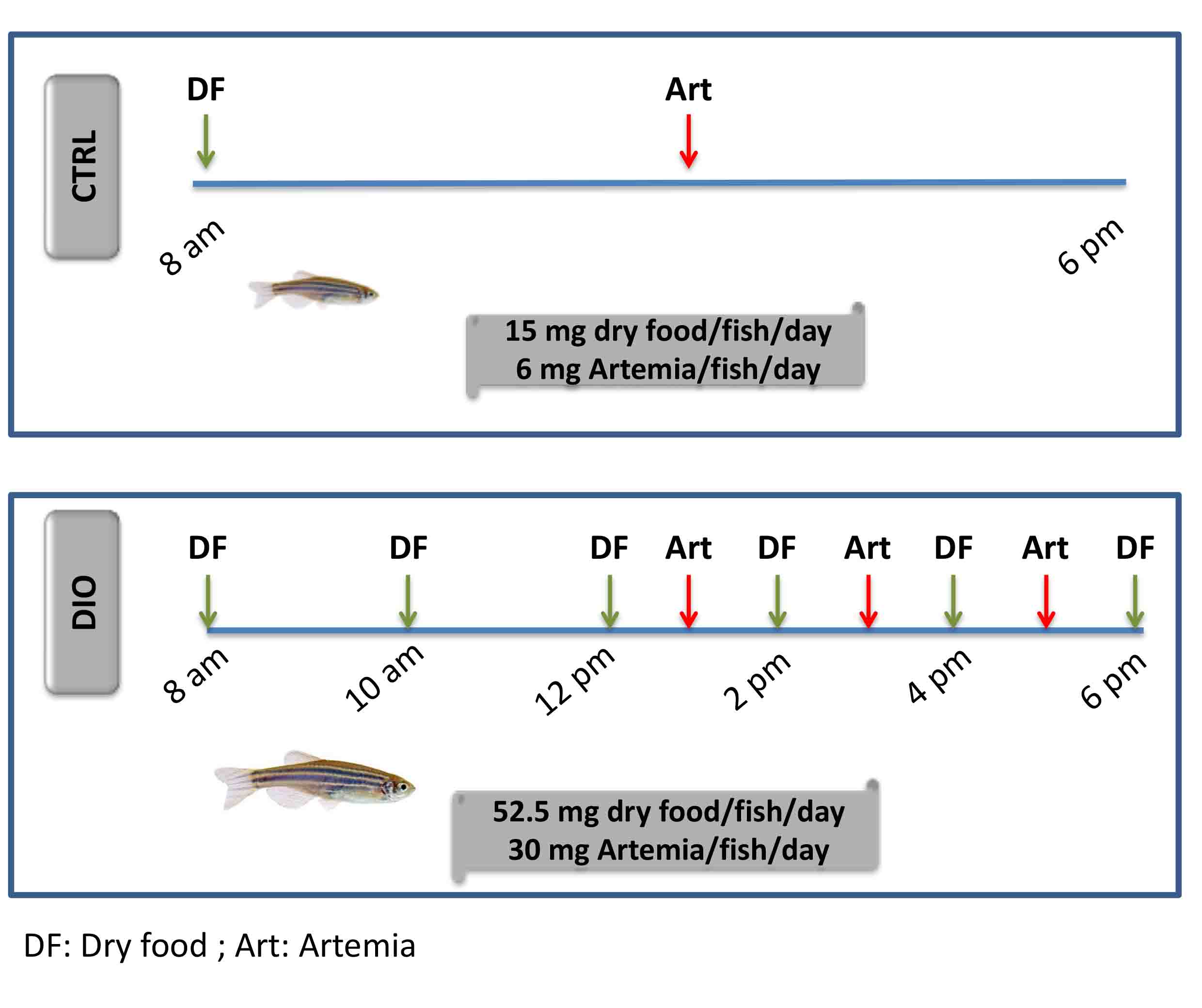
**

**Suppl. Fig 1: Feeding protocol in control (CTRL) and overfed fish (DIO)**

**
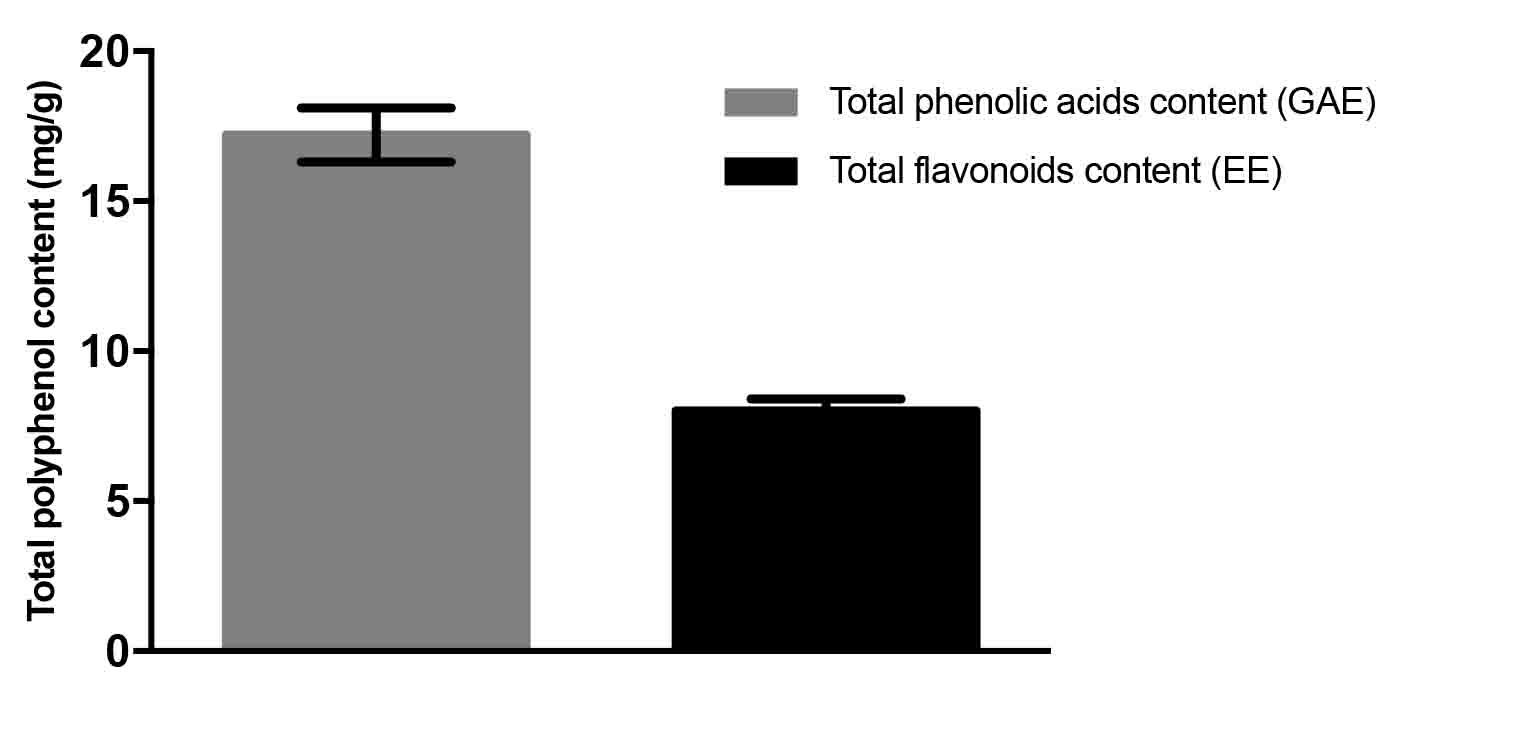
**

**Suppl. Fig. 2: Total polyphenol content of *A. borbonica* infusion.**

Phenolic acids and flavonoids levels were determined by using colorimetric assays and expressed as mg gallic acid equivalent (GAE) / g plant dry powder or mg epicatechin equivalent (EE) / g plant dry powder, respectively.

**
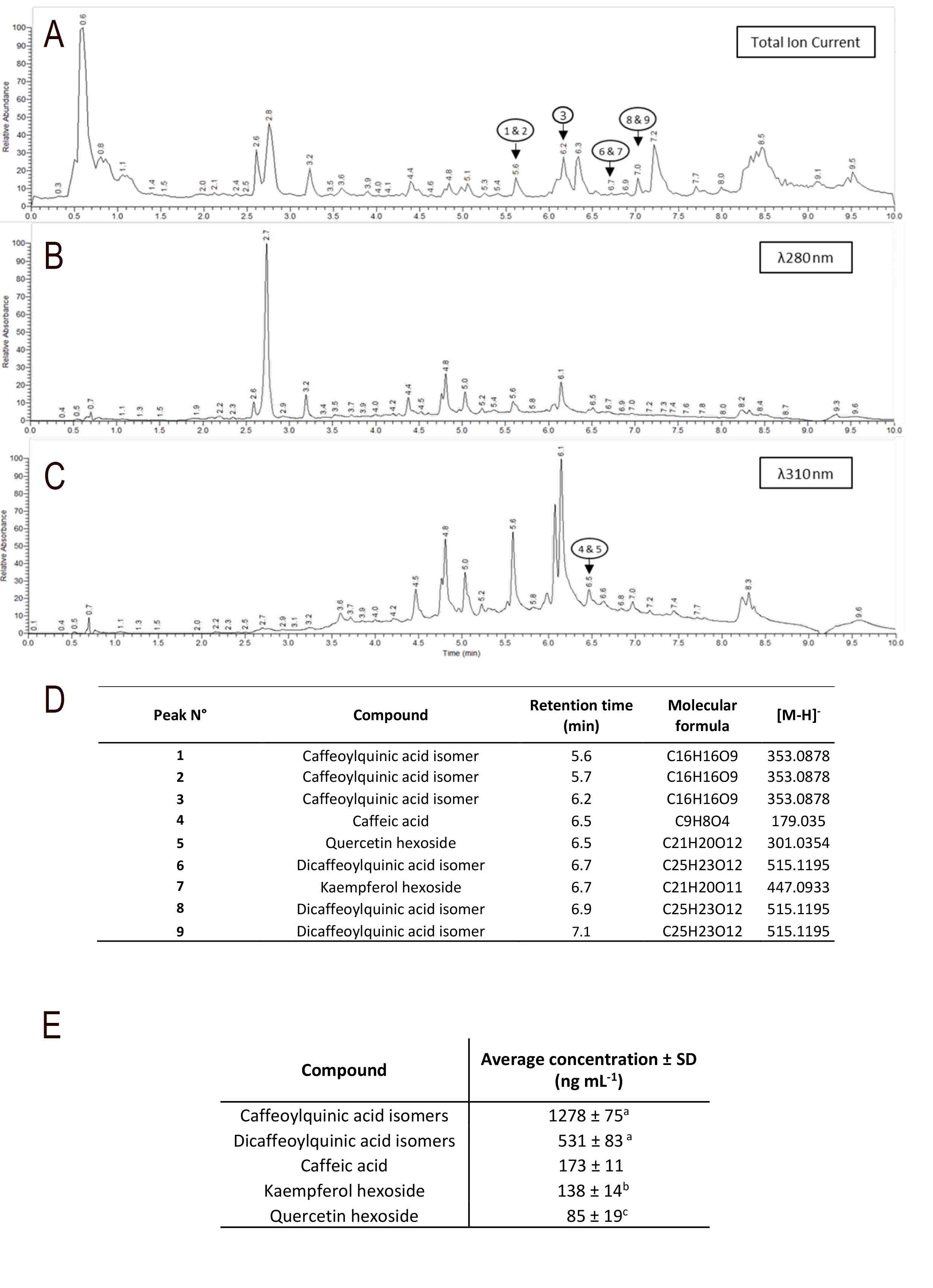
**

**Suppl. Fig. 3: Identification of polyphenols from *A. borbonica* infusion by LC-UV-ESI-MS.**
**A:** Representative total ion current. **B:** Representative UV spectrum at 280 nm. **C:** Representative UV spectrum at 310 nm. **D:** Identification of the peaks indicated in the spectrum in A and B, with the name of the compounds, its formula, retention time and mass [M-H]. **E:** Average concentration of the compounds identified by mass spectroscopy.

Calculated concentrations from caffeic acid ^(a)^, Kaempferol ^(b)^, Quercetin ^(C)^ calibration curves, respectively.
